# Amplification of Snake Venom Toxicity by Endogenous Signaling Pathways

**DOI:** 10.3390/toxins12020068

**Published:** 2020-01-22

**Authors:** Philip E. Bickler

**Affiliations:** 1 Department of Anesthesia and Perioperative Care, University of California at San Francisco, San Francisco, CA 94143-0542, USA; philip.bickler@ucsf.edu; 2 California Academy of Sciences, San Francisco, CA 94118, USA

**Keywords:** phospholipase A2, metalloprotease, snake venom, intracellular signaling, neuromuscular paralysis, intracellular calcium

## Abstract

The active components of snake venoms encompass a complex and variable mixture of proteins that produce a diverse, but largely stereotypical, range of pharmacologic effects and toxicities. Venom protein diversity and host susceptibilities determine the relative contributions of five main pathologies: neuromuscular dysfunction, inflammation, coagulopathy, cell/organ injury, and disruption of homeostatic mechanisms of normal physiology. In this review, we describe how snakebite is not only a condition mediated directly by venom, but by the amplification of signals dysregulating inflammation, coagulation, neurotransmission, and cell survival. Although venom proteins are diverse, the majority of important pathologic events following envenoming follow from a small group of enzyme-like activities and the actions of small toxic peptides. This review focuses on two of the most important enzymatic activities: snake venom phospholipases (svPLA_2_) and snake venom metalloproteases (svMP). These two enzyme classes are adept at enabling venom to recruit homologous endogenous signaling systems with sufficient magnitude and duration to produce and amplify cell injury beyond what would be expected from the direct impact of a whole venom dose. This magnification produces many of the most acutely important consequences of envenoming as well as chronic sequelae. Snake venom PLA_2_s and MPs enzymes recruit prey analogs of similar activity. The transduction mechanisms that recruit endogenous responses include arachidonic acid, intracellular calcium, cytokines, bioactive peptides, and possibly dimerization of venom and prey protein homologs. Despite years of investigation, the precise mechanism of svPLA_2_-induced neuromuscular paralysis remains incomplete. Based on recent studies, paralysis results from a self-amplifying cycle of endogenous PLA_2_ activation, arachidonic acid, increases in intracellular Ca^2+^ and nicotinic receptor deactivation. When prolonged, synaptic suppression supports the degeneration of the synapse. Interaction between endothelium-damaging MPs, sPLA_2_s and hyaluronidases enhance venom spread, accentuating venom-induced neurotoxicity, inflammation, coagulopathy and tissue injury. Improving snakebite treatment requires new tools to understand direct and indirect effects of envenoming. Homologous PLA_2_ and MP activities in both venoms and prey/snakebite victim provide molecular targets for non-antibody, small molecule agents for dissecting mechanisms of venom toxicity. Importantly, these tools enable the separation of venom-specific and prey-specific pathological responses to venom.

## 1. Introduction

The World Health Organization (WHO) estimates that snakes envenom about 400,000 people per year, causing more than 148,000 deaths. Permanent disabilities such as amputations, wound contractures, and functional loss of limbs are often the result of non-fatal envenoming [1,2]). In total, 5.8 billion people live directly in or within an hour of venomous snake habitats, putting nearly three-quarters of the world’s population at risk [3].

Current standard of care therapy for snakebite is focused on the use of antibody-based treatments (here termed “serotherapies”) intended to intercept, neutralize and remove venoms present in the circulation before they produce long-term effects [4]. The basic concepts and methodology underlying serotherapy were developed more than a century ago: stimulated by Pasteur’s and Behring’s work on rabies and diphtheria, Albert Calmette produced antisera effective against cobra venom [5]. Vital Brazil was the first to develop polyvalent antisera against South American snakes and described the chief component pathologies of envenoming: coagulopathy, hemolysis, cytotoxicity, and paralysis [6]. Attempts to understand the mechanisms of venom toxicity began even earlier, with Fayer’s seminal recognition in the 1860s of the physiological similarities between the actions of cobra venom and the plant toxin curare [7].

Proteomic science has revealed that snake venoms are a diverse and variable mixture of enzymatic and non-enzymatic proteins and peptides. Venom complexity presents several important challenges to the understanding of venom mechanisms. The most obvious challenge is dissecting the separate effects of venom components in an envenomed animal. A summary of the major known venom toxicities and mechanisms, classified by key effects, molecular nature (enzymatic or non-enzymatic), timing of action, and sites of effect, is presented in Table 1. A more detailed compendium of venom components was recently published by Waheed et al. [8]. Laustsen states that snake venom is the “most complex pharmaceutical target” known, composed not only of a multitude of toxin components but a multitude of biochemical interactions [9].

Snakebite is not only a condition mediated directly by venom proteins but also by the reaction of the body to the venom. Some of the ways that venom engages endogenous signaling systems in prey or victim are included in the far-right column of Table 1. Based on convergent biochemical and physiological information, it has been possible to resolve that the most important effects of venom are based on a handful of, rather than many, molecular effectors. This small number of endogenous processes have an outsized effect on the regulation of cell function and cell survival. Co-opting endogenous processes enables venoms to use the envenomed organisms’ own cellular machinery to disrupt neurotransmission, dysregulate coagulation, and produce mediators of inflammation.

The foci of this review are hypotheses related to the manner in which venoms enhance their lethality by co-opting prey signaling systems, disordering and amplifying the prey’s inflammation and cell survival machinery. Identifying key venom targets and their endogenous counterparts for inhibition should be applicable to most medically important snake species, and simultaneously address the fundamental matter of the recipient’s biological response to the venomous insult.

## 2. Enzymatic and Non-Enzymatic Components of Venom and Where They Act

**Figure 1:**
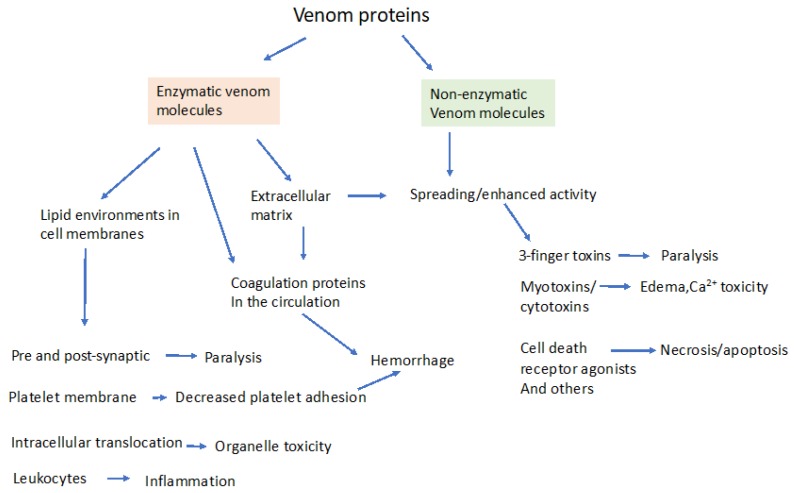
General targets of major snake venom proteins divided into venoms that have intrinsic enzymatic activity and those that are non-enzymatic. Enzymatic venom proteins are typically hydrolases such as PLA_2_, serine proteases, metalloproteases, or hyaluronidases, releasing biologically active products that act on the extracellular matrix, on membrane proteins, on membrane-based signaling molecules or inside cells. Examples of non-enzymatic venom components include the curare-like 3-finger toxins from kraits, potassium channel blocking dendrotoxins and pore-forming myotoxins. Enzymatic destruction of the extracellular matrix by metalloproteases and hyaluronidases enhance venom spread and amplify toxicity. Other, direct acting, non-enzymatic protein toxins no doubt exist in yet to be characterized venoms. Further, venom proteins may simultaneously have enzyme-based and non-enzyme-based toxicities, such as components of PLA_2_ heterodimers, blurring these distinctions. Considerable cross-talk between enzymatic and non-enzymatic venom components may exist, for example non-enzymatic svPLA_2_s may dimerize and activate endogenous catalytic PLA_2_ proteins [18].

Venom proteins can be broadly classified into those that contain intrinsic enzymatic activity and those that do not. The obvious significance of this distinction is the possible inhibition of the enzymatic venom components by small molecule therapeutic agents. Proteomic analysis reveals that snake venoms contain proteins from 26 protein families, with substantial species variation [28,29,30]. However, the most prevalent medically relevant components occur in just four families, in varying proportions [10,31]. These proteins are: (1) secreted phospholipase A_2_ (sPLA_2_); (2) metalloproteases (MPs); (3) serine-proteases (SPs); and (4) the non-enzymatic three-finger toxins (3-FTX, e.g., α-bungarotoxin, an antagonist of nicotinic receptors [28,31]). Not all snake venom toxins are so neatly classified, including dendrotoxins from mambas, which are competitive antagonists of voltage gated potassium channels, and myotoxins from Crotalids that create cation permeability channels in the sarcolemma [24,25,32]. The main actions of these groups of enzymatic and non-enzymatic venom components are shown in Figure 1. It is important to note that some quantitatively minor venom components can have outsized effects on venom toxicity; for example, hyaluronidase from *Crotalus durissus terrificus* represents only 0.23% of the total protein, but greatly potentiates crotoxin lethality [33].

Molecular understanding of venom toxicity, based on enzymatic and non-enzymatic actions, developed slowly. The first advance in the modern era was the recognition by Karl Slotta and Heinz Fraenkel-Conrat in 1938 that crotoxin crystalized from *Crotalus durissus* was a phospholipase [34]. Another leap occurred in the 1970s when it was established that α-neurotoxins are competitive nicotinic receptor blockers [21,35]. Gutierrez and Lomonte have published a valuable review of seminal developments in the field [18].

Venoms typically act quickly to immobilize prey, with non-lethal doses more slowly producing weakness and a dose-dependent range of tissue and organ toxicity. Few venoms cross the blood–brain barrier, or even gain access to the extravascular compartment environment without assisted vascular leakage. Instead, to exert biological effects, larger or enzymatic venom proteins either: (1) bind to other proteins in the body (e.g., α-toxins); or (2) enzymatically create small molecular mass signaling molecules that have spatially and pharmacologically broader effects. Based purely on molecular mass considerations, svMPs (we use the terms svMP and svPLA_2_ to designate snake venom metalloproteases and phospholipases A_2_ to distinguish those enzymes from the secreted or intracellular enzymes present in prey/victim.) are expected to have effects confined to the circulation. However, the actions of svMPs yield small molecular mass peptides that are both biologically active and spread quickly. Phospholipases are smaller proteins and gain earlier access to deeper compartments in the body, where they generate cell-specific signals. Excluding for the moment direct protein–protein interactions in the extracellular compartment (e.g., proteases that hydrolyze coagulation proteins), these biological effects include:

(1) Production of mediators that diffuse within or across cell membranes;

(2) Production of transmembrane signals by direct binding to cell surface receptors such as neurotransmitter receptors/ion channels or G-protein coupled receptors;

(3) Translocation into the cell via transporters, carriers or endocytosis.

Any molecular description of venom effects must also account for the variability and time-dependent pathology seen in both lethal and sub-lethal envenoming. These effects can be diverse, even when caused by envenoming by a single species or closely related group of snakes. For example, in a recent review, Frare and colleagues described the delayed and variable clinical manifestations of *Crotalus durissus* envenoming. The summarized clinical reports describe muscle and kidney damage, neurotoxicity and hemolytic/coagulopathic pathologies to varying degrees [36,37,38]. These variations in clinical presentation may be related to differences in venom composition and effects that vary with season, locality, and even with sub-populations of the same species of *Crotalus* [39].

### 2.1. Venom Translocation from Circulation to Interstitial Compartment

Based on current knowledge, venoms rely on penetration of barriers rather than specific transport mechanisms to leave the circulation. Relatively little is known about the rate of spread, extent of distribution, and persistence of venom proteins in the body. Knowledge concerning the distribution of venom in the circulation, based on pharmacokinetic models and published information, was summarized recently by Sanhajariya [40]. The conclusion of Sanhajariya’s review was that the limited knowledge about the pharmacokinetics and pharmacodynamics of venoms restricts our understanding of venom-venom and whole venom–host interactions. Since the effects of envenoming can be long-lived, it is of interest to know whether venom persistence or host-response persistence explains long-lasting toxicity.

One of the ubiquitous weapons in venom is metalloproteases [41]. These enzymes attack basement membranes and collagen in tissues to increase venom spread and disrupt blood vessels [14]. Importantly, the digestion products of metalloproteases can be biologically active: these bioactive peptides affect tissue growth, remodeling, repair and development [42]. In addition, these bioactive peptides increase venom toxicity by triggering and amplifying inflammation [15]. Further, they induce the transcription and translation of endogenous matrix metalloproteases that attack types of collagen that are outside the range of svMPs [41].

Hyaluronidases are also important in venom spread and represent a nearly ubiquitous venom component [12,27]. Hyaluronidase activity amplifies the toxicity of crotoxin by enhancing venom distribution. In one study, *Crotalus durissus terrificus* crotoxin injected into mice was only toxic when co-injected with purified hyaluronidase [33]. Other mechanisms of hyaluronidase toxicity involve bioactive products of the enzymatic digestion of hyaluronic acid [13].

### 2.2. Venom Binding or Interaction with Cell Surface Receptors

There are several lines of evidence that venoms bind or interact with cell surface proteins. However, clear demonstration of venom binding/incorporation into membranes or binding to a particular acceptor/receptor was a challenge for many years. While α-neurotoxins were known to bind with high affinity to nicotinic acetylcholine [21], there was no similar demonstration of a “receptor” for β-neurotoxins. The nature of weak membrane interactions of PLA_2_ was explored in the 1970s and 1980s, identifying only low affinity interactions [43]. Studies by Oberg and Kelly [44] used iodine-125 labeled-β-bungarotoxin to identify a class of membrane fragments associating with the toxin in rat brain. Binding sites were found in cell membrane and mitochondrial fractions but the studies did not characterize the affinity of binding or whether the binding sites were protein, carbohydrate or lipid. In the 1970s, McDermott et al. used [^3^H]-pyridoxylated β-bungarotoxin to identify binding in synaptosomes and synaptic vesicles from rat brain. Binding occurred at relatively low affinity to a protein “acceptor” that was distributed widely in several membrane fractions, including synaptic vesicles [45].

By the 1990s, it was becoming accepted that the neuro- and myotoxic sPLA_2_ interact with specific receptors or interacting proteins (reviewed by [46]). One of the clearest demonstrations of such specificity is the high-affinity cell surface binding of PLA_2_s from the venom of *Oxyuranus scutellatus*. Different components of *Oxyuranus* venom bind specifically to neuronal and muscle membranes [47]. Additional evidence for selectivity is the accelerated evolution of PLA_2_ genes, first clarified by the work of Nakashima and colleagues in the 1990s [48]. The model proposed by Kini and Evans [49] in 1989 explained the fundamental aspects, if not the molecular details, of this amazing diversity and specificity the of toxic activities of venom PLA_2_s.

As mentioned, it is clearly established that one class of venom proteins, the α-neurotoxins (3FTx), bind with high affinity to nicotinic acetylcholine receptors at the neuromuscular junction (NMJ) [50]. Nicotinic receptors rendered non-functional by α-bungarotoxins are eventually de-phosphorylated, a state that identifies them for internalization [50]. Internalized receptors are then degraded by the proteasome complex.

### 2.3. Venom Transport into the Intracellular Compartment

Evidence from several recent studies suggests that one of the ways venom proteins may cause intracellular effects is to physically translocate into the cytosolic compartment. For example, Lagonder and colleagues demonstrated the translocation of a β-neurotoxin (gold-labeled mutant ammodytoxin A) into the of terminal axon and terminal boutons of a mammalian motor neuron. Interestingly, the labelled toxin was not found in the muscle fibers themselves [51]. In contrast, myotoxic PLA_2_ was demonstrated to be exclusively found on the sarcolemma. Several additional demonstrations of internalization of β-neurotoxins into various neuronal cells have also been published. In one study, the fluorescent protein label Alexa was used to label notexin, β-bungarotoxin and taipotoxin. These labeled proteins were found to localize to mitochondria in rat cerebellar granular neurons and spinal cord motor neurons [52]. Vimentin, a ubiquitous filament protein that anchors organelles within the cytoplasm, has also been identified as associating with venom PLA_2_. Vimentin binding may both facilitate PLA_2_ hydrolytic activity and facilitate internalization of the catalytic subunit of PLA_2_ [46]. A recent report [53] found evidence of intracellular location of three types of snake venom PLA_2_s, based on elegant protein labeling studies in myotube cultures. Adding to this body of evidence, Sribar’s team suggested that venom proteins with PLA_2_ activity may be transported into the pre-synapse [54].

## 3. Molecular Effectors in Elapid and Viper Venoms

We will next review the main enzymatic activities of snake venom proteins. These primary enzymatic effects are frequently amplified within the prey, broadening and prolonging the pathology.

### 3.1. Snake Venom Phospholipases (svPLA_2_)

**Figure 2:**
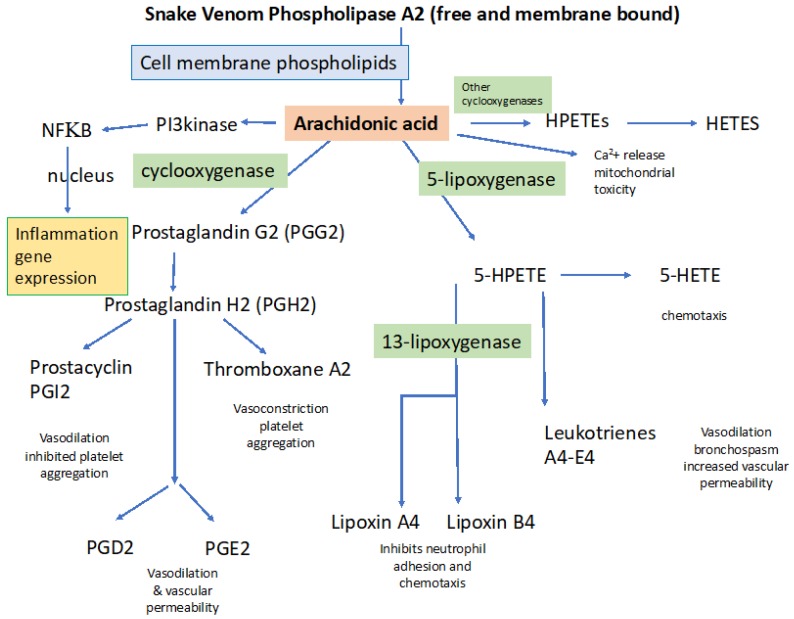
Arachidonic acid metabolism stimulated by snake venom phospholipases. The primary effect of svPLA_2_ is production of arachidonic acid. Direct effects of arachidonic acid include activation of the transcription factor NFκΒ, responsible for the transcription of numerous genes encoding cytokines, release of intracellular Ca^2+^ from the endoplasmic reticulum, and phosphorylation of intracellular kinases. Arachidonic acid is also metabolized by cyclooxygenases and lipoxygenases, producing prostaglandins and leukotrienes [57]. Once set in motion, the inflammatory cascade is thus diversified and amplified by additional signaling molecules.

Phospholipase enzymes are found in nearly all forms of life including bacteria, plants, invertebrates and vertebrates. PLA_2_s have roles in the regulation of phospholipid turnover and membrane lipid content. Their most important physiological role is the production of arachidonic acid (AA). AA is the first step in the production of eicosanoids, leukotrienes and prostaglandins (Figure 2).

Vertebrate PLA_2_s comprise a large superfamily of enzymes composed of 16 recognized groups within six major types, as reviewed by Harris and Scott-Davey [46] (particularly see references 1–6 therein): These major types include the secreted PLA_2_s (sPLA_2_), the cytosolic PLA_2_s (cPLA_2_), the calcium independent PLA_2_s (iPLA_2_), the platelet activating factor (PAF) acetyl hydrolase/oxidized lipid lipoprotein associated PLA_2_s (LpPLA_2_s), the adipose PLA_2_ (AdPLA_2_s) and the lysosomal PLA_2_s (LPLA_2_s). Extracellular PLA_2_ requires millimolar to micromolar [Ca^2+^] for full activity, whereas the intracellular PLA_2_ is active in the nanomolar level Ca^2+^ range that characterizes the intracellular environment.

As is true of all PLA_2_s, svPLA_2_s catalyze specific hydrolysis of the ester linkage at the sn-2 position of glycerophospholipids. The catalytic site for the generation of AA lies in a grove accessible on the surface of the venom protein. However, PLA_2_-like proteins found in snake venoms may be devoid of catalytic activity. These non-catalytic subunits may exhibit myotoxic, neurotoxic, or pro-inflammatory effects [55,56]. The mechanisms of non-catalytically active PLA_2_ toxicity are poorly understood.

Snake venom PLA_2_s are probably the most pharmacologically active, multi-effect venom components [46]. Phospholipases are found to varying degrees in virtually all snake venoms and in the saliva of non-venomous and minimally venomous snakes as well, with a notable preponderance in the venoms of Elapid snakes. The catalytic components of svPLA_2_s principally act by hydrolyzing the glycerol ester of membrane bound arachidonic acid, liberating free arachidonic acid (AA). Arachidonic acid is highly reactive and stimulates important pathways that govern a myriad of biological processes. Arachidonic acid stimulates the creation of a family of biologically active signals, including: (1) members of the cyclooxygenase pathway, involving prostaglandins and thromboxane; (2) members of the lipoxygenase pathway, forming leukotrienes; and (3) regulation of the Cytochrome P450 group of enzymes, including lipoxygenases that form hydroperoxy-eicosatetraenoic acids (HPETEs) and hydro-eicosatetraenoic acids (HETEs). A summary of the role of these signaling molecules following snake envenoming with notations as to how they contribute to venom pathology (light blue boxes) is shown in Figure 2.

svPLA_2_s exist as monomers, dimers, heterodimers, trimers and hexamers composed of varying combinations of catalytic and non-catalytic subunits. The detailed functions of most non-catalytic units are still poorly defined, but some are thought to be involved in trafficking the catalytic unit to specific tissues, and in some cases direct cytotoxicity. This basic concept of PLA_2_ toxicity was first incorporated into a model by Kini and Evans about 30 years ago [49], although the enzymatic and non-enzymatic potential of PLA_2_ heterodimer components was not known at the time. It is now clear that formation of multi-unit complexes of catalytic and non-catalytic PLA_2_s can dramatically alter toxicity. In a remarkable demonstration, in vitro dimerization of catalytic and non-catalytic subunits from different families of snakes can produce enhanced toxicity: dimerization of crotoxin-A and a single β-chain of agkistrodotoxin increased toxicity over monomers [58]. For a review of the importance of venom component complexes to toxicity, see Doley and Kini [59].

svPLA_2_s have, by far, the broadest pathologic effects of any snake venom proteins. An excellent historical perspective and review of the development of knowledge about the role of PLA_2_s in venom toxicity was written by Gutierrez and Lomonte, recounting progress from the first crystallization of venom PLA_2_ to recent molecular understanding of PLA_2_ action [18]. Snake venom PLA_2_s are directly responsible for early- and late-onset symptomology, as well as synergistic and regulatory roles for other snake venom components [60]. sPLA_2_s are intimately involved in the peripheral neuro-myotoxicity caused by envenoming bites of many dangerous snakes and because both s- and cPLA_2_ are implicated in inflammatory and degenerative disease of the nervous system, roles that are discussed below. PLA_2_ can also mediate cell-based toxicity, for example in *Bothrops* PLA_2_ myotoxicity [61]. PLA_2_s can be the dominant venom component in some species. A recent paper from Calvete’s group showed that 60% of the proteins in Russell’s viper belong to the PLA_2_ family [62]. The widespread distribution of PLA_2_ in snake venoms suggests a universal potential for toxicity involving the dysregulation of processes involving arachidonic acid.

Intracellular PLA_2_ activity must liberate arachidonic acid from membranes inside the cell, having powerful actions. Importantly, this would include release of Ca^2+^ from intracellular sites of sequestration, mediated by arachidonic acid and activation of ryanodine receptors [63]. Because membrane bound PLA_2_ generates intracellular arachidonic acid, and intracellular action of PLA_2_ does not, in principle, require translocation of sPLA_2_ into the intracellular compartment.

One of the most interesting and important features of svPLA_2_s is their capacity to activate PLA_2_ homologs in the tissues of prey, in a process of homologous protein activation. This results in an amplification of the effects of svPLA_2_ beyond that of the venom alone. This mechanism also provides a relay of toxicity by non-catalytic svPLA_2_s to endogenous catalytic PLA_2_ proteins. A transcellular relay of phospholipase activity was first described by Shier who reported experiments in 1979 showing activation of endogenous PLA_2_ by a fraction of cobra venom [20]. This action, mediated by cobra “Lytic factor”, seems to be specific to activation of endogenous intracellular PLA_2_. Further, purified mellitin, which contains no intrinsic PLA_2_ activity, greatly increases intracellular PLA_2_ activity in cultured cells. These key results were among the first to show that venom can relay protease activity to the intracellular compartment, co-opting the cells own machinery. This relay and amplification indicate that venom PLA_2_s can have effects that are larger than would be guessed based on their percentage distribution in venoms.

Because of its near ubiquity in snake venoms and clinically significant effects, sPLA_2_ is an appealing candidate for inhibition by small molecule therapeutics [64] and recent studies have demonstrated that PLA_2_ activity of a diverse group of venoms can be inhibited by the agent varespladib [65,66,67,68]. Varespladib was recently shown to bind to both catalytic and non-catalytic PLA_2_s, reducing myotoxicity mediated solely by a non-catalytic PLA_2_ [69].

### 3.2. Snake Venom Metalloprotease (svMP)

Metalloproteases are large molecular mass proteins, >50 kDa. Metalloproteases are a family of proteases that were originally grouped by their requirement for divalent cations (zinc and cobalt) for full activity. Subsequent studies revealed important diversity and subgroups based on structure, substrate and regulatory control [70]. Simply by virtue of their size, these enzymes act predominately in the circulatory compartment, playing roles to facilitate the dispersion of smaller molecular weight venom components (e.g., phospholipases) and in signaling and amplifying the toxicity of other venom components. It is important to note that metalloproteases are distinct from matrix metalloproteases (MMPs), although there is a key interaction, via inflammation, and induced gene expression by the digestion products of both classes of proteases [71,72].

It is generally accepted that metalloproteases in snake venoms play central roles in hemorrhage, by loosening the connective tissue components responsible for blood vessel structural integrity [41]. The pioneering histologic studies of McKay and Owenby [73,74] described destruction of basement membranes of capillaries in tissues exposed to hemorrhagic venom components, actions now thought primarily to be mediated by these metalloprotease enzymes. The ability of svMPs to degrade various types of extracellular matrix proteins has been demonstrated in vitro by protein electrophoresis and immunoblot techniques, allowing visualization of digestion fragments. svMPs hydrolyze laminin, nidogen, enactin, type IV collagen, fibronectin, and proteoglycans (for review see [41]). A recent proteomic analysis revealed an even greater range of protein targets [75]. Although metalloproteases have numerous effects in vitro that support a causative role in bleeding, the relative contribution of svMP to hemorrhage in vivo has been difficult to clearly ascertain for several reasons. First, other components of snake venoms, such as phospholipases and serine proteases, inhibit or alter coagulation proteins [8]. Second, tools such as selective small molecule inhibitors to isolate svMP effects from matrix metalloproteases have only recently been available. Examples of metalloprotease inhibitors include prinomastat and marimastat, developed to inhibit cancer metastasis [76].

The mechanism of action of hemorrhagic svMPs involves cleavage of structurally important basement membrane components. This includes type IV collagen, followed by the mechanical disruption of vessels due to hemodynamic biophysical forces (i.e., a “two-step” process, see [41]). The identification of the regions in the molecular structure of svMPs that determine their ability to bind to the cleavage sites of basement membrane proteins are incompletely known. Studies by Gutierrez and Fox indicate that fragments of extracellular matrix proteins and other types of proteins released in the tissues as a consequence of svMP action may be normally involved in the processes of tissue repair and regeneration [41]. svMPs have a synergistic effect with PLA_2_ activity, related to these protein fragments. The complexity of potential interactions between PLA_2_s and MPs in producing tissue damage has recently been reviewed [77].

A number of observations suggest that *endogenous* proteases contribute importantly to the structural damage caused by snake venom structural proteases (for review [41]). Matrix metalloproteases are synthesized and secreted by resident and infiltrating cells in the course of the inflammation that follows tissue damage by venom. A further important effect of metalloproteases is that their action initiates and supports ongoing inflammation. Evidence for this action derives from studies in which relatively pure venom metalloprotease preparations have been injected into rodents and immune responses quantified. A number of such studies have reported increases in interleukins, PGE_2_, TNF-α, as well as changes in leukocyte populations and migration from the action of svMPs [78,79,80]. For a recent review of svMPs and immune modulation, see the review of Burin et al. [17]. Additional studies are needed to clarify the mechanism of immune modulation by metalloproteases, whether it is direct signaling by cleavage products or simply exposure of basement membranes and tissue factors during the digestion of the extracellular protein matrix.

### 3.3. Snake Venon Hyaluronidases

Although not present in large quantitites in the venom of any known snakes, hyuronidases apparently act to amplify the toxicity of other venom components by increasing the rate and spread of the injected toxins [12]. The products of proteoglycan hydrolysis produced by hyaluronidases have biological activity as well, with hyaluronan fragments participating in the acute pharmacological effects of envenoming, including the inflammatory response, by upregulating matrix metalloproteases [81]. This interesting body of work is reviewed by Kemparaju et al. [13]. Inhibition of venom hyaluronidase activity with natural or synthetic compounds has been a subject of several recent studies [12,82].

### 3.4. Other Directly Cytotoxic Proteins

A number of snake venom proteins have been reported to be directly cytotoxic in cell culture. Some venom proteins create cation channels in cell membranes, flooding cells with sodium and calcium and producing cell death by calcium intoxication, ionic imbalance and gross water movement/swelling/cell rupture. For review, see Waheed et al. 2017 [8]. Some of these toxicities include non-catalytic components of PLA_2_ heteromers.

Death receptors DR4 and DR5 are cell membrane proteins that trigger apoptosis. It is unclear if this is a direct binding or the consequence of paracellular or intracellular signals generated that activate these receptors via phospholipase cascades. Death receptor DR4 and DR5 may be activated by some types of venom, e.g., *Vipera lebetina* [26], but the interaction of venom and these receptors is not yet well characterized. Apoptosis is an actively mediated form of cell death and would seem not to be part of the immediate threat of envenoming. Apoptosis involves complex intracellular machinery and is an example of how a venom co-opts the prey’s own signaling apparatus to kill cells.

### 3.5. Non-Enzymatic 3-Finger Toxins (3FTx, -Neurotoxins)

3FTx proteins are found predominately in elapid venoms. In king cobras (*Ophiophagus hannah*) and Eastern green mambas (*Dendroaspis angusticeps*), 3FTx proteins make up about 70% of the proteins in venom [83]. In desert coral snakes (*Micrurus tschudii*), the proportion is reported as high as 95% [84]. This group of snake venom toxins are generally high affinity competitive antagonists of nicotinic acetylcholine receptors, making them one of the few groups of venom proteins whose action is well characterized. The diversity of 3FTs and the genomics of these proteins were recently reviewed [85]. High affinity binding of α-bungarotoxin to nAChRs is widely employed in receptor biology to label and study nAChRs.

## 4. Prey Response to Venoms: How Venoms Co-Opt Signaling Pathways to Produce Toxicity

Snakebite is not only a condition mediated directly by venom proteins but by the reaction of the envenomed animal to the venom. Understanding secondary causes of venom toxicity is a challenge to designing effective therapies to combat the serious delayed effects of envenoming in initial snake bite survivors.

### 4.1. Inflammation and Inflammation-Mediated Cytotoxicity

#### 4.1.1. General Processes Involved in Activating Inflammation: Importance of PLA_2_

In view of the diversity of venom proteins, it is perhaps not surprising that snake venoms are capable of activating all pathways of the immune response. A novel proposal relating to immune reactions to snakebite is that type II immune responses evolved in vertebrates to protect against venom. This hypothesis was advanced by Galli and colleagues [86]. Type II immune responses usually involve acquired immunity, mediated by IgE antibodies and mast cells, producing seemingly inappropriate immune responses like anaphylaxis to foods and other common antigens. Mast cells can degranulate following envenoming even with no prior specific immunization or sensitization. The resulting intense humoral immune response protects against otherwise fatal venom exposures [86,87]. However, the interaction of the immune system and snake venoms is more complex than just type II responses. As highlighted by Burin and colleagues [17], snake venom accomplishes both immune suppression and immune stimulation, with the pattern of reaction to venom snake species-specific. The potential of using these actions of venom for immune therapy and for understanding immune responses has been much discussed [88].

A particularly broad and complex venom-induced inflammatory response is seen after cobra envenoming, with both pro- and anti-inflammatory components [89]. *Naja annulifera* venom triggers acute systemic inflammation, including PLA_2_-dependent neutrophilia and increased plasma levels of IL-6 and MCP-1. In mice, 2LD_50_ dose of *Naja* venom caused both neutrophilia and monocytosis. In an in vivo experimental model in mice, Silva-de-Franca et al. [90] found that *Naja annulifera* venom induced swelling and several histopathologic changes in the hind paws of the animals. In addition, myonecrosis associated with inflammation was observed, an event that is commonly found in experimental models of elapid envenoming. This was attributed to a cytotoxic action of some PLA_2_ component.

Nearly all snake venoms initiate and sustain pathological inflammation in the body [91,92]. The consequences of this pathological inflammation include both short- and long-term effects of tissue damage and organ system failure. The first demonstration of an inflammatory reaction to snake venom seems to be that of Damerau et al. who in 1975 showed that cobra venom lytic factor caused mast cell degranulation, histamine release and cytokine production [93]. Brain and Whittle in 1977 reported that the inflammatory actions of Russell’s viper PLA_2_ generates a dose-dependent release of histamine. Brain and Whittle also believed that the venom released or enhanced endogenous PLA_2_ [94]. Recent studies have coalesced on the concept that svPLA_2_ and svMP appear to be the most important venom components in producing inflammation [16,95]. svPLA_2_ and svMP activities sidestep the usual foreign protein recognition aspect of immune activation to directly stimulate immune cells, via arachidonic acid and pro-inflammatory digestion products [17]. This creates a response disproportionate to the immediate direct immunogenicity of the venom proteins. PLA_2_ activity in venom produces high levels of arachidonic acid and related inflammatory cytokines both in serum and inside cells. Activation of innate immune cells by venoms triggers a self-amplifying cascade of pro-inflammatory cytokine production, including IL-6, TNF- and IL-1, chemokines, and lipid mediators, which produce a positive feedback loop of leukocyte migration and activation. Lipid mediators include eicosanoids derived from the arachidonic acid metabolism, such as prostaglandins, leukotrienes, and thromboxanes. These mediators, in combination with cytokines and chemokines, trigger various clinical and pathologic features of inflammation, including edema, pain, chemotaxis, cytokine release, and leukocyte activation. In addition, hyaluronidase, glycosylated proteins and svMPs promote different aspects of inflammation, which includes a range of effects, including complement activation [15].

We speculate that *endogenous* intracellular PLA_2_ recruitment in immune cells increases the venom’s ability to create a robust humoral immune response. The proposed mechanism involves arachidonic acid, ryanodine receptors, intracellular calcium, protein kinase C, endogenous PLA_2_ and calcium release from intracellular reservoirs, a universal mechanism involved in PLA_2_ signaling [63]. Activation of endogenous PLA_2_ is also probably involved. An example is in streptococcal bacterial infections in the lungs. PLA_2_-like proteins from these bacterial recruit and activate endogenous PLA_2_s in pulmonary macrophages and lung parenchyma [96]. A similar pattern of injury mediated by PLA_2_ in muscle tissue has been described [16]. Mediation of inflammatory pathways by metalloproteases is also likely to contribute to these effects [90].

In the above examples, the primary toxicity caused by svPLA_2_ is amplified and prolonged by the recruitment of endogenous PLA_2_. As with other venom effects, the contributions of primary and prey-enhanced inflammatory responses remain to be fully defined. Modern techniques to track systemic mediators of inflammation (cytokine arrays) could be employed to assess these events, as could cytokine mRNA arrays and the use of small molecule inhibitors.

#### 4.1.2. Inflammation Underlies Increased Vascular Permeability and Tissue Edema

Inflammation produced in response to envenoming has the potential to affect every organ in the body. Inflammation-mediated fluid extravasation into tissue, when profound, can lead to tissue edema and systemic hypovolemia and circulatory shock. Longer-term effects of inflammation can lead to cell death and organ failure.

Inflammation-induced pulmonary edema is a common pulmonary complication of snakebite. Other impacts on the respiratory system include pulmonary hemorrhage [97] and, obviously, neuromuscular paralysis and respiratory failure [98]. Pulmonary edema following envenomation is most likely related to capillary leak mediated by inflammatory mediators [99], although cardiogenic pulmonary edema can occur as well [100].

Systemic inflammation and circulating cytokines such as TNF-α following envenoming may be associated with venom-induced lung injury in humans. In different models of hemorrhagic shock, plasma, pulmonary and hepatic increases in IL-6 and MCP-1 are observed with inflammation and lung injury, which may culminate in acute respiratory distress syndrome [101]. It is important to emphasize that in addition to cytokines, some other venom proteins such as enzymes that attack basement membranes (metalloproteases and hyaluronidases) may produce pulmonary hemorrhage [8].

### 4.2. Coagulation Disorders

Bleeding and disordered coagulation is seen with many types of envenoming, suggesting strong natural selection for venom proteins that produce coagulopathy. Coagulopathy following envenoming is complex, with contributions from svPLA_2_s, svMPs, hyaluronidases, serine proteases, and others [8]. Because phospholipids serve as potent co-factors for numerous enzymatic conversions in the intrinsic and extrinsic clotting cascades, it is not surprising that phospholipases play a potent role in regulating or disrupting coagulation. Venoms, through their PLA_2_ activity, achieve potent, multi-site anticoagulation by co-opting just a few key processes that regulate coagulation. Accordingly, venom PLA_2_ is a prime target for therapeutic intervention in the coagulopathy of snakebite [2,8,90,102].

#### 4.2.1. Specific Role of svPLA2 in Disordered Coagulation

Dysregulated platelet adhesion, mediated by svPLA_2_, is a significant component of the pathology of snake-venom-induced coagulation disorder. This was apparently first suggested by Boffa and Boffa in the 1970s, and was presented model form by Kini and Evans [49]. PLA_2_-dependent effects include inhibition of platelet adhesion, release of thromboxane, serotonin and adenosine diphosphate. Each of these platelet-derived factors contribute importantly to disordered coagulation. The initiating event in producing all these mediators is the PLA_2_-dependent production of arachidonic acid from phospholipids in the platelet membrane or in circulating lipoprotein particles. The importance of PLA_2_ in coagulopathy was neatly demonstrated by the neutralization of the coagulotoxic effects of *Naja* venom with the specific PLA_2_ inhibitor varespladib [102]. Similar PLA_2_-dependent coagulation disruption was demonstrated by Anilkumar et al. [103] with a series of imidazopyridines that inhibit PLA_2_ activity in Russell’s viper venom.

#### 4.2.2. Role of svMPs in Hemorrhage

Venom metalloproteases and venom-activated prey matrix metalloproteases are important in hemorrhage. It is not clear whether svMP activity is entirely direct, or whether its action also sets in motion other signaling molecules such as bioactive protein digestion products, expression of tissue factor and coagulation and expression of endogenous metalloproteases, in an amplification cascade. Current evidence reveals a very complex interrelationship between svMPs and endogenous matrix metalloproteases (MMPs). Gutierrez and colleagues [41] note that the degradation of some types of fibrillar collagen after envenoming depends upon the action of endogenous matrix metalloproteases (MMPs) in prey. Endogenous MMPs are rapidly expressed in prey as a result of the induction of inflammation mediated by svPLA_2_ and activation of endogenous PLA_2_. svMPs generate biologically active proteins from the hydrolysis of proteins in the extracellular matrix. For example, hydrolysis of types XV and XVIII collagen generates endostatin, an angiogenesis inhibitor. The cleavage of the α-3 chain of type IV collagen by matrix MPs releases tumustatin, another antiangiogenic molecule. This complex mixture of biologically active fragments of extracellular matrix degradation serves to amplify and broaden the initial effects of the svMPs. Therefore, the interaction of inflammation and protease action on the extracellular matrix and blood vessel integrity is crucial in determining the pathologic effects of venoms containing svMPs. These topics have been extensively explored because of the implications for inhibition of angiogenesis in cancer treatment [76]. Only recently has research moved more broadly to consider the effects of svMPs other than effects mediating structural damage to basement membranes including those of blood vessels. Disruption of microvessel integrity is accepted as the most important cause of hemorrhage caused by viperid venoms [14]. The expression of matrix MPs and tissue inhibitors of MPs by cells is regulated by numerous cytokines (particularly interleukin-1, IL-1), growth factors and hormones, some of which are specific to cell type and others that are ubiquitous (e.g., transforming growth factor beta, TGF-beta) [76]. Many of these factors are products of monocytes/macrophages and their production in inflammatory situations is therefore part of the chain of events leading to tissue degradation. Tissue destruction, both physiological and pathological, is correlated with an imbalance of inhibitors and activators [42]; snake venoms would likely tip the scales.

### 4.3. Paralysis

**Figure 3:**
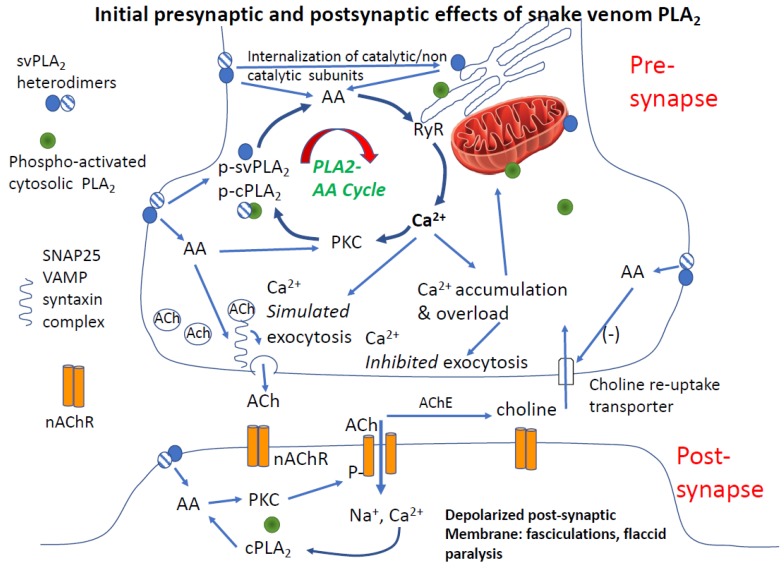

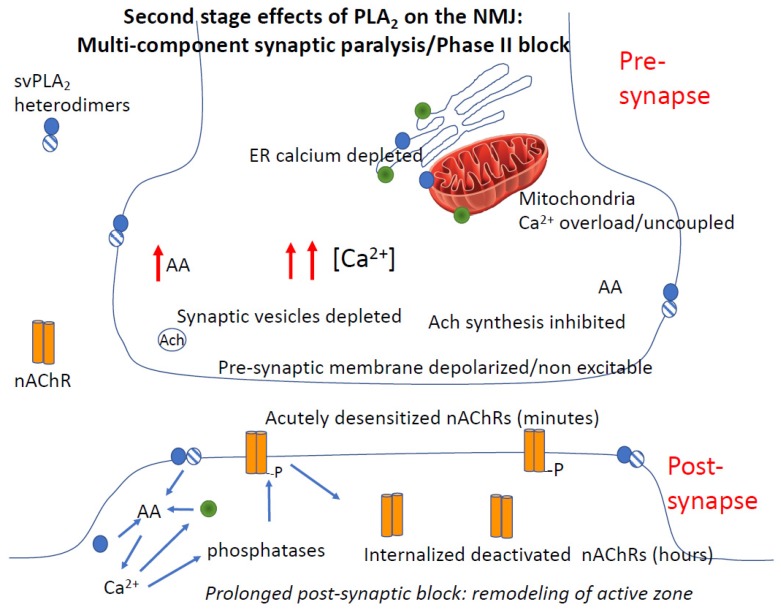
Multi-site failure of synaptic transmission mediated by svPLA_2_s. **Upper panel** shows cycle of amplification of arachidonic acid and calcium signaling causing rapid depletion of pre-synaptic acetylcholine vesicles, increases in intracellular Ca^2+^ [Ca^2+^]_i_ and acute desensitization of post-synaptic nicotinic acetylcholine receptors. Key events include snake venom (svPLA_2_)-mediated increase in pre-synaptic arachidonic acid (AA), and increases in pre-synaptic [Ca^2+^]_i_ from release from intra-neuronal stores in the endoplasmic reticulum and augmented by voltage-gated calcium channels (not depicted). These actions are amplified by direct AA activation of protein kinase C, which facilitates activation of the vesicle fusion protein complex. Both catalytic and non-catalytic PLA_2_ subunits (shaded and cross-hatched circles, respectively) are potentially able to co-activate endogenous PLA_2_. Activation of intracellular, endogenous, PLA_2_ is part of the amplification cycle. The net effect is depletion of pre-synaptic transmitter vesicles and mitochondrial Ca^2+^ uptake. AA inhibition of the choline re-uptake transporter amplifies the decrease of releasable acetylcholine. **Lower panel** depicts the short and longer-term effects of PLA_2_ at the neuromuscular junction. Following the initial burst of acetylcholine release, post-junctional acetylcholine receptors are desensitized and then inactivated (dephosphorylated, internalized) analogous to their state in a phase II neuromuscular block produced by large/repeated doses of succinylcholine. As in the pre-synapse, PLA_2_ mediates a self-amplifying cycle of increase in arachidonic acid, intracellular calcium, and calcium-sensitive phosphatase activation. The process is augmented both by internalization of svPLA_2_ and/or activation of endogenous PLA_2_. The post-synaptic membrane is now depolarized and unexcitable for a prolonged period.

Paralysis is one of the most dramatic and consequential effects of envenoming. However, other than the well-defined actions of 3FTxs (e.g., α-bungarotoxin) as competitive antagonists of nicotinic acetylcholine receptors, one of the least understood. Paralysis of skeletal muscles, including respiratory muscles, is the cardinal effect of elapid venoms but also a clinical feature of many types of viper envenoming. A very strong body of evidence points to PLA_2_ activity in venom (i.e., β-neurotoxins) as the main cause of paralysis produced by bites from some of the most medically important snakes in the world. Among the clinically relevant effects of β-neurotoxins are depletion of pre-synaptic neurotransmitter vesicles, inactivation of nicotinic acetylcholine receptors and eventual physical degeneration of the neuromuscular endplate. Ranawaka and colleagues recently reviewed some of the controversies related to PLA_2_ paralysis, from the perspective of the actions of krait β-neurotoxin [104]. An integrated two-part model for how we believe PLA_2_ mediates a multi-target attack on neuromuscular transmission is shown in Figure 3. The initial effects of facilitation, then rundown of acetylcholine release is depicted in the upper panel, and inactivation of transmission at the post-junction are shown in the lower panel. Together, these effects explain both the rapid and sustained effects of PLA_2_ at the neuromuscular junction.

#### 4.3.1. Clinical Features of Paralysis from β-Neurotoxins

There are several features of the paralysis produced by PLA_2_ venoms that must be accounted for in any mechanistic explanation. The first is that fasciculations (myokymia) frequently precede or accompany the onset of clinical weakness. In contrast, fasciculations are never seen in neuromuscular block produced by competitive antagonists of nicotinic acetylcholine receptors, e.g., the curare-like non-depolarizing agents used to produce muscle relaxation for surgery or critical care [105]. Fasciculations also are never seen even during subclinical block or during block recovery from non-depolarizing agents. However, fasciculations are a clear feature of drugs that produce depolarizing block, such as succinylcholine or decamethonium, or presynaptic toxins such as botulinum or nerve agents. As a result of facilitated acetylcholine release, these agents cause both depletion of pre-junctional neurotransmitter and depolarization of the post-junctional membrane, at least for a period of time. Fasciculations are a clinical sign of disordered release and accumulation acetylcholine, inhibited disposal of acetylcholine in the neuromuscular junction, or activation of extra-junctional receptors. Fasciculations have been clearly described in bites involving PLA_2_ venoms, in both elapids [106] and vipers [107,108].

Fasciculations precede and cause a second and very important (although short-lived) phenomenon at the neuromuscular junction: desensitization of post-junctional nicotinic acetylcholine receptors. Acute desensitization was one of the first autoregulatory aspects of nicotinic transmission at the neuromuscular junction described by Sir Bernard Katz in seminal studies of neuromuscular junction function [109]. The details of this have been revealed in the frog neuromuscular junction to involve the action of calcium-dependent negative feedback inhibition of neurotransmission mediated by pre-synaptic nicotinic and muscarinic autoreceptors [110]. At the same time that desensitization of post-junctional nicotinic receptors is occurring, depletion of synaptic vesicles develops, such that nerve depolarization does not accomplish neurotransmission. This pre-synaptic effect is based on the antecedent enhanced vesicle fusion and release, Ca^2+^ entry, and Ca^2+^ facilitation of phospho-activation of the vesicle release protein system. Basically, the neurotransmitter system is depleted and not replenished (see Figure 3).

The third effect is seen at the post-junctional component of the NMJ. When desensitization is accompanied by post-synaptic increases in [Ca^2+^], calcium-sensitive phosphatases are activated and nicotinic receptors undergo the process of inactivation. As this persists, receptors are removed from the synapse in a process of cytoskeleton-mediated internalization. This is a long-lasting effect. The pharmacology of receptor desensitization and inactivation is detailed in a review by Albuquerque [50]. This action by β-neurotoxins toxins is more speculative, although it is a mechanism based on many studies of nicotinic receptor regulation in various preparations [111]. It is important to note that there is some evidence that PLA_2_s can act as competitive nAChR antagonists, similar to the better defined α-neurotoxins [112]. The quantitative importance of this α-effect is unresolved.

The final observation that must be accounted for in a mechanistic explanation is that, following very prolonged paralysis mediated by β-neurotoxins, physical damage to the synaptic structure may occur. A model of PLA_2_ toxicity must also account for the observation that relatively long periods of neuromuscular block (4–6 h) can be reversed by small molecule PLA_2_ inhibitors. Such a rescue was in swine envenomed with *Micrurus* venom was observed with the PLA_2_ inhibitor varespladib [67].

#### 4.3.2. Is PLA_2_ and Arachidonic Acid Sufficient to Cause Synaptic Failure

Not excluding other mechanisms, we believe that arachidonic acid generated by svPLA_2_ is sufficiently potent to account for the clinical and pathologic features outlined above. A key mechanism is that AA generated by catalytic svPLA_2_s or endogenous PLA_2_s potently stimulates Ca^2+^ release from the endoplasmic reticulum [54], accounting for the initial surge of acetylcholine release and fasciculations. Increased Ca^2+^ in the pre- and post-junctional compartments are amplified by other mechanisms as well, including Ca^2+^ entry through voltage-gated calcium channels. Observed electrophysiological effects of crotoxin are consistent with these actions. For example, both crotoxin dimer (one catalytically active and one catalytically inactive PLA_2_ subunit) and the basic PLA_2_ subunit monomer, have a biphasic and calcium-dependent effect on nerve-evoked transmitter exocytosis. A transient initial facilitation followed by a sustained decay of transmitter release, is observed. Monomer and dimer reduce nerve-evoked radiolabeled-ACh release by 60% and 69%, respectively, but only the crotoxin heterodimer decreased the amplitude of nerve-evoked muscle twitches [19]. This model is also consistent with in vitro studies of *C. durissus* crotoxin [113]. Experiments of neuromuscular junction electrophysiology and intracellular Ca^2+^ measurements and small molecule inhibitors of secreted PLA_2_s would be very helpful in confirming or refuting the validity of the role of Ca^2+^ in these interpretations.

Augmenting pre-junctional failure, AA inhibits the choline reuptake transporter, contributing to the depletion of pre-synaptic terminals of releasable acetylcholine [114]. Combined with the other presynaptic actions of arachidonic acid, blockade of choline re-uptake is a significant contributor to paralysis. These actions comprise pre-synaptic focus of PLA_2_ toxin effects, consistent with electrophysiology done in the seminal work of Chang et al. [115]. However, technically speaking the work of Cheng did not rule out some blockade at the post-synapse, just not a block of the muscle itself, such as a prolonged muscle depolarization or toxicity to myofibrils.

Arachidonic acid also interacts with SNARE proteins that regulate neurotransmitter release, contributing to the biphasic effects of β-neurotoxins on neuromuscular transmission. This produces an initial facilitation followed by a long-lasting depression of neuromuscular function. This effect most likely involves both vesicle depletion and receptor inactivation as discussed above; i.e., both pre- and post-synaptic inhibition of NMJ function [116]. Consistent with our model, the initial effects of svPLA_2_ increase transmitter release in an exuberant and uncontrolled manner, and then produce deactivation of post-junctional receptors.

Arachidonic acid mediates release of Ca^2+^ from intracellular stores such as the endoplasmic reticulum; these alterations can be cytotoxic, particularly in the context of other co-occurring cellular stress [117].

#### 4.3.3. Post-Junctional Effects of PLA_2_ Venoms

In the post-synapse, AA activates protein kinase C (PKC) [118]. Activated PKC phosphorylates nicotinic receptors, increasing their activity but contributing rapidly to desensitization, which is a conformational state of the receptor protein which renders it less capable of activation by acetylcholine [112]. A second and key stage of receptor inactivation occurs with receptor de-phosphorylation by calcium-sensitive phosphatases, resulting in prolonged internalization of these now inactivated nicotinic receptors. Inactivated receptors can remain intact in the intracellular compartment of the cell for 5 or more hours, before being re-inserted into in the post-synaptic membrane as functional receptors. This is a period of profound synaptic inactivation [119]. In addition to functional changes in the synapse, internalization of nicotinic receptors causes cytoskeletal-dependent structural changes in the synaptic structure. Thus, PLA_2_, via AA, co-opts machinery crucial to the function of both the pre- and post-synapse in the neuromuscular junction, producing a multidimensional, profound and persistent blockade of neuromuscular function.

PLA_2_ or closely associated proteins have been proposed to directly antagonize nAchRs [113], similar to a-toxins. This does not seem to us to be a complete explanation for several reasons. First, the initial clinical presentation of PLA_2_-induced paralysis does not fit that of a competitive antagonist (see above) or explain the initial facilitation of neurotransmitter release seen in electrophysiology studies. Also, there is no demonstrated high-affinity of -neurotoxins for nicotinic receptors.

#### 4.3.4. Role of Calcium in PLA_2_ Mediated Pre- and Post-Junctional Block of Neurotransmission

The cycle of effects initiated by PLA_2_ forms a positive feedback loop involving plasma membrane PLA_2_, endogenous PLA_2_, Ca^2+^ release, kinase activation and activation of Ca^2+^ release/influx by multiple mechanisms (upper panel in Figure 3). This process is not unique to snake venoms, but a widespread mechanism clearly described in other cells [63]. One of the most important effects of the AA-Ca^2+^ amplification cycle is grossly elevated intracellular Ca^2+^. A study by Tedesco neatly demonstrated this action: the large and sustained increases in interterminal [Ca^2+^] produced by a snake PLA_2_ neurotoxin (β-bungarotoxin, taipaitoxin) was similar to that of the well-characterized black widow venom α-latrotoxin [120].

*Bothrops asper* myotoxins type I and II induce Ca^2+^ release from inside the cell, most likely from the endoplasmic reticulum, which contains by far the largest amount of Ca^2+^ inside cells [121,122,123]. This is also observed in human brain endothelial cells, where arachidonic acid releases intracellular Ca^2+^ by inositol triphosphate and ryanodine receptors. This work is convergent with our earlier work showing that release of calcium from the ER is a key player in adaptation to hypoxia and in ischemic preconditioning in neurons [124]. In addition, triggered release of Ca^2+^ by venom is a plausible mechanism of cellular toxicity, and would involve mitochondrial dysfunction caused by mitochondrial Ca^2+^ overload, as in neurodegenerative diseases [125]. The neurotoxic secreted phospholipase A_2_ from the *Vipera a. ammodytes* venom targets cytochrome-c oxidase in neuronal mitochondria [54].

#### 4.3.5. PLA_2_ and Rapid Degeneration of the Synapse

A key question is whether venoms permanently destroy neuromuscular junctions or cause potentially reversible physical changes in the structure and function of the junction. Current dogma posits rapid destruction of the pre-synapse [126]. It has been known for some time that -neurotoxins such as bungarotoxin produce physical changes in the structure of motor nerve terminals that precede axonal degeneration of motor nerves [127]. Indeed, rat muscles paralyzed with β-bungarotoxin show loss of synaptic vesicles, changes in mitochondria, loss of boutons and other changes and ultrastructural changes observed between 3 and 6 h. However, the hypothesis that the morphological changes observed within 3–6 h represent degeneration (and presumed loss of rescue potential) do not fit with the observation that PLA_2_ inhibitors can reverse induced antivenom resistant paralysis in swine caused by *Micrurus* venom some 4 h after envenoming [67]. Thus, the hypothesis that β-neurotoxins cause irreversible damage to the NMJ soon after envenoming may need modification.

Structure and function of the neuromuscular junction are intimately linked. The neuromuscular junction is highly plastic, changing anatomical form with function, and requiring constant input from neurotransmission and trophic factors for its health and permanence. Nicotinic receptor activation state is a key part of this regulation, because receptors are connected to the cytoskeleton. When receptors are inactivated, they are internalized, and the cytoskeleton complex that determines the shape of the NMJ is altered at the same time. Neuromuscular junction function and morphology both can change over the course of minutes [128]. These cytoskeletal features are highly conserved across different animals, with similar mechanisms in mammals, insects and mollusks [129]. Accordingly, neuromuscular block, regardless of mechanism, leads to reversible changes in synaptic structure. Some of the changes in synaptic morphology seen in ER studies could represent structural changes due to receptor internalization, without actual cell degeneration. This possibility widens the therapeutic window for reversing neuromuscular block induced by venoms, consistent with late rescue of neuromuscular dysfunction by varespladib [67]. Thus, β-neurotoxins may be more like nerve agent toxins than botulinum toxin in their effects on the neuromuscular junction, at least in terms of reversibility, if not mechanism. Certainly, persistent paralysis can cause atrophic degeneration; this is also seen in the curare-like drugs used for long periods (even in humans with botulism or prolonged neuromuscular block in the intensive care unit [130]).

## 5. Interaction and Amplification of Venom MPs and PLA_2_s in Inflammation and Coagulation

**Figure 4:**
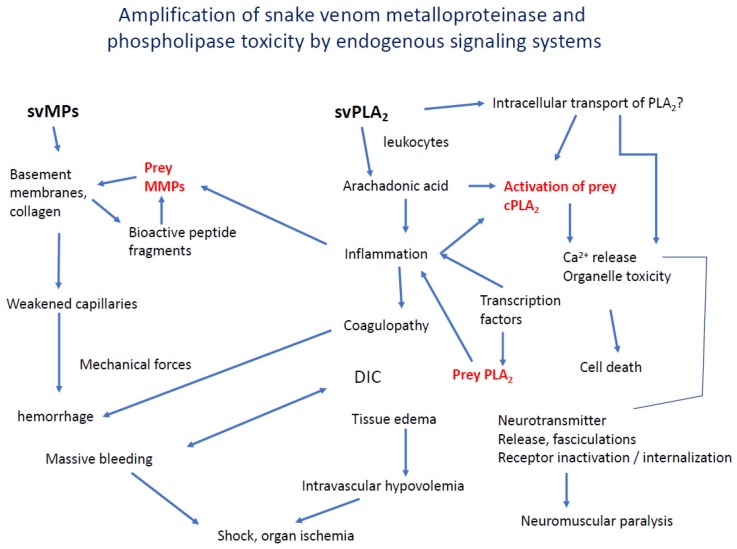
Interaction of the effects of enzymatic venom components to produce and amplify immediate and long-term toxicity for immobilizing prey and deterring predators. cPLA_2_, cytosolic/endogenous PLA_2_s. cPLA_2_ = cytosolic/endogenous PLA_2_. MMP = endogenous, inducible matrix metalloproteases, DIC = disseminated intravascular coagulation.

We have described some ways that svMPs and svPLA_2_s and probably other venom components act synergistically, mediated by their biologically active products. Teixeira and colleagues have written an excellent recent review of the convergence and interaction of inflammation and coagulation disorders in snake envenoming [88]. More work is needed to uncover these important interacting mechanisms and to discover therapeutic strategies based on them. Figure 4 is a synthesis of known and speculative pathway convergence.

## 6. Tools Needed to Dissect the Multi-Compartment Action of Snake Venoms

The hypothesis that venom mechanisms involve a substantial contribution from prey response can be tested with a number of tools and approaches. These tools include: (1) small molecule selective inhibitors of specific signaling components; (2) genetic models that have been engineered for deletion or amplification of specific signal elements; (3) assay of prey gene expression or cytokine expression profiles, either alone or in combination with #1 and #2. None of these approaches have been extensively used to understand reaction to snake venoms.

In contrast, antivenom serotherapy has a limited capacity to dissect venom mechanisms. Serotherapy is a treatment that predominately intercepts venom molecules in the plasma or lymph. Whether polyvalent or monoclonal, antibodies are mostly restricted to the blood and perhaps to a limited degree to the interstitial compartment (i.e., antiserum has a small volume of distribution) and this is also a limitation of their utility as research tools for dissection of venom mechanisms.

## 7. Areas Where New Knowledge Concerning the Cellular Effects of Venom Is Needed

The complexity of snake venoms has clearly deterred efforts to formulate molecular understandings of venom action. However, we are optimistic that progress towards such an understanding is possible. This optimism is supported by evidence that small molecule drugs, acting at specific enzyme sites, can rescue late toxicity, such as in the case of the PLA_2_ inhibitor varespladib [67,68,69]. Other highly selective small molecule inhibitors have the potential to reveal previously unknown, or underappreciated, components of venom toxicity, and venom-initiated endogenous responses.

Detailed understanding of the processes involving co-option of endogenous pathways and processes will contribute to new therapeutic directions for snakebite treatment. Several of the outstanding questions related to these endogenous components are presented in Table 2. Addressing the hypotheses in the Table will uncover host-based pathological responses to venom and suggest the advantages and disadvantages to different therapeutic approaches. Ultimately, whether the dissection approach is genetic, proteomic, or biochemical pathway specific, systems need to be understood in their functional totality. The World Health Organization’s recent attention to priority actions on snakebite therapeutics has directed attention to some of the newer approaches.

## 8. Conclusions

Snake venoms rely on proteases and small toxic peptides to elaborate signals that co-opt universal vertebrate signaling pathways that regulate coagulation, inflammation, neuromuscular function, and cell survival. Venoms act in the circulation, in the intracellular compartment, within cell membranes, and inside cells. Just a few types of protease activities underlie the capabilities of venom to derail regulation in the PLA_2_ and MP pathways, making it likely that small molecule inhibitors of PLA_2s_ and MPs will provide synergistic therapeutic benefit. In addition, because these small molecules are not restricted in distribution as is current serotherapy, they have a potentially much wider therapeutic window for late effects of venom proteins. A major gap in our understanding is whether the intracellular effects of venom are due to intracellular translocation of venom protein including non-catalytic subunits of svPLA_2_s, or second messenger effects from arachidonic acid, Ca^2+^, etc. A further gap in our knowledge is whether the benefits of drugs like varespladib are due to inhibition of venom protease activity, inhibition of prey protease activity, or a mixture of the two effects.

Recent advances in testing and development of small molecule inhibitors of snake toxins and snake toxin effects take us into new territory in several important ways. First, they provide rescue therapy beyond what antivenoms can do. Second, they provide insight into the physiology and pathology of snakebite in ways that antivenoms never could. Because of the exquisite selectivity of new agents against venom protease activities, we can begin to unravel how snake venoms not only exert direct toxicity, but co-opt reactive inflammation in the prey/victim to produce toxicity to organs, cause paralysis, and other short and long-term effects.

## Figures and Tables

**Table 1:** Main snake venom components, grouped by broad effects (structural mechanisms light green, coagulation yellow, paralysis grey, cardiovascular/cell signaling orange, and cell toxicity light blue). The percent contribution of each venom component varies; PLA_2_ and MP components predominate in many venoms. Also presented are the chief mechanism of effect and time course, and whether toxicities involve subversion of the envenomed animal’s homeostasis regulating machinery. Classification timing of action reflects: (1) Rapid; immediate (less than a few minutes) in blood compartment: does not require translocation or second messengers; (2) Intermediate (minutes to an hour): requires generation of second messenger signals and translocation outside circulation; and (3) Delayed (initiated or persistent for hours to days): requires extensive translocation and slower acting/regulated events such as apoptosis. Abbreviations: ECM extracellular matrix.

Venom Component	Primary Pathologic Effect	Site of Action	Timing of Effect	Enzymatic or Non-Enzymatic	Venom Action Amplified by Prey
Disintegrins	Inhibit cell-ECM, loosen anchoring tissue [10]	Interstitial spaces	Intermediate and late?	Non	Yes, augments inflammation [11]
Hyaluronidases	Loosens tissue, enhances venom spread [12], exposes tissue factor	Capillaries and Interstitial spaces	Intermediate	Enzymatic	Yes, augments inflammation, coagulopathy [13]
Metalloproteases	Loosens/digests basal lamina [14]	Capillaries connective tissue	Intermediate	Enzymatic	Yes, bioactive peptides [15], gene expression
Serine Proteases	Inhibit coagulation, anti-thrombin effect [8]	Blood	Rapid	Enzymatic	Yes, signal cascades
Antithrombins	Hydrolysis of thrombin, clot destabilizer [8]	Blood	Rapid	Enzymatic	Unknown
PLA_2_s (inflammation, coagulation)	Production of arachidonic acid, mediators of inflammation [16,17]	leukocytes, platelets, endothelial cells	Rapid	Enzymatic and non-enzymatic subunits [18]	Yes, Ca^2+^, arachidonate, phosphorylation, gene expr. [17]
PLA_2_s (-neurotoxin)	Paralysis	Neuromuscular junction [19]	Usually rapid but may evolve slowly	Enzymatic and non-enzymatic	Yes, homologous protein activation [20]
3FTx (-neurotoxins)	Paralysis/anticholinergic	Antagonists of nicotinic/muscarinic receptors [21]	Rapid	Direct	No
Cysteine-rich secretory proteins (CRISPS)	Target ion channels, Ca^2+^ release	Endothelium, leukocytes [22]	Delayed	unknown	Unknown
Kallikrein-like proteins	Shock, physiological disturbance [8]	Vasodilator	Rapid	Direct	Yes, amplifies inflammation
Phosphodiesterases	Hydrolysis of cyclic nucleotides/cell signaling/vasodilation [23]	Cell membrane, intracellular	Intermediate	Enzymatic	Yes, cell signaling pathways
Myotoxins	Cell damage [24,25]	Sarcolemma	Intermediate Delayed	Direct	Possible, overlap/identity to some PLA_2_s
Activators of cell death receptors DR4 and DR5	Programmed cell death (apoptosis) [26]	Liver, kidneys, muscle	Delayed	Direct	Yes, cell apoptosis machinery
L-Amino Acid Oxidases	Free radicals tissue damage, immune activation [27]	Blood, extracellular fluid	Intermediate	Enzymatic	Yes, cytokine gene expression

**Table 2:** Important questions and hypotheses concerning the relative roles of direct or recipient-endogenous components in snake venom toxicity. Included is evidence supporting or in opposition to each question or hypothesis. The column on the right lists some newer models or experimental tools that could be used to test these hypotheses or generate new questions. MMP = endogenous matrix metalloproteases, SMT = small molecular therapeutics, EM = electron microscopy, NMJ = neuromuscular junction, ER = endoplasmic reticulum.

Key Question/Hypothesis	Best Evidence for	Evidence Against	What Is Needed?
Is the catalytic action of svPLA_2_ responsible for paralysis?	Fasciculations [107], Pre-junctional effects, Ca^2+^ measurements [54], arachidonic acid	EM of altered NMJ morphology Post junctional effects [127]	Electrophysiology calcium imaging knockout mice. Small molecule inhibitor studies
Is endogenous PLA_2_ activation required for paralysis by β-toxins?	Small molecule inhibitors of endogenous PLA_2_ reverse paralysis [67,68]	None, excluding possible α-toxin effects of some PLA_2_s [113]	Ca^2+^-chelators, ER calcium imaging/release inhibitors should block synaptic failure
Do β-toxins cause *reversible* changes in the NMJ?	Delayed rescue by SMTs possible [67,68]	Ultrastructural images of damage [127]	Small molecule inhibitor studies, longer term assessment of NMJ structure
Is recruitment of prey PLA_2_ required to initiate or sustain inflammation?	Inflammation is sustained for long duration, failure of serotherapy to address [4]	No specific evidence against	Small molecule inhibitors selective for endogenous PLA_2_, cytokine gene arrays, genetic models
Can inhibition of endogenous PLA_2_ prevent organ toxicity?	Not studied	Organ damage caused by non PLA_2_ venom components [8]	Assessment of renal, hepatic, pulmonary function. Small molecule inhibitor studies
Are svPLA_2_s and svMPs synergistic in producing inflammation?	Elevations of cytokines, expression of MMPs [17]	Evidence lacking	Cytokine gene arrays with and without SMTs Cytokine levels
